# Quantification of nickel, cobalt, and manganese concentration using ultraviolet-visible spectroscopy†

**DOI:** 10.1039/d1ra03962h

**Published:** 2021-08-19

**Authors:** Monu Malik, Ka Ho Chan, Gisele Azimi

**Affiliations:** Department of Chemical Engineering and Applied Chemistry, University of Toronto 200 College Street Toronto Ontario M5S 3E5 Canada g.azimi@utoronto.ca; Department of Materials Science and Engineering, University of Toronto 184 College Street Toronto Ontario M5S 3E4 Canada

## Abstract

Ultraviolet-visible spectroscopy is one of the most effective, inexpensive, flexible, and simplest analytical techniques to measure species concentration in the liquid phase. It has a wide range of applications such as wastewater treatment, dye degradation, colloidal nanoparticle characterization. It is used in almost every spectroscopy laboratory for routine analysis or research. In the present study, a feasibility study was carried out to find the application of UV-Vis spectroscopy for onsite measurement of nickel, cobalt, manganese, and lithium as a replacement for the conventional method to measure the concentrations of these elements in battery and other applicable industries. Samples with different concentrations of individual elements and composites were prepared and analyzed using an ultraviolet-visible spectrometer. Based on the obtained results, mathematical relationships between concentration and absorbance were defined. The calculated concentration of different elements using the developed relationships was compared with the measured concentration using ICP-OES to find any deviation between the two. The effect of various parameters such as concentration, path length, number of elements in the solution, density, and pH was analyzed to verify the feasibility. The obtained results show that this technique can be effectively used to measure the concentration of nickel and cobalt with high accuracy.

## Abstract 2

Ultraviolet-visible spectroscopy is one of the most effective, inexpensive, flexible, and simplest analytical techniques to measure species concentration in the liquid phase.

## Graphical abstract

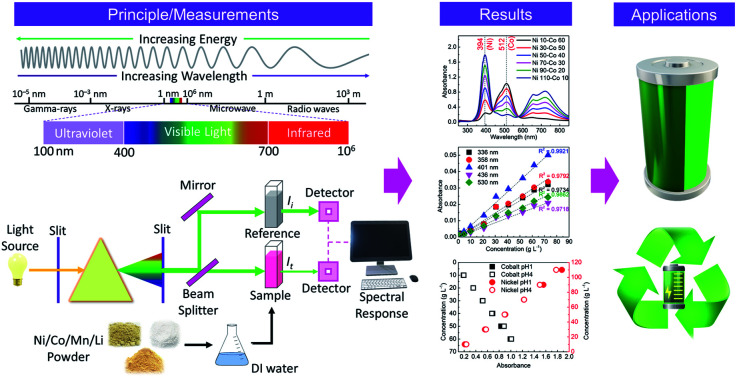

## Introduction

Currently, the measurement of nickel (Ni), cobalt (Co), manganese (Mn), and lithium (Li) concentration in various industries such as battery production, battery recycling, and ore separation is achieved using various laboratory-based analytical techniques such as inductively coupled plasma optical emission spectroscopy (ICP-OES).^1–3^ These measurements are primarily carried out for process control, which involves the movement of a sample from a processing environment to a centralized laboratory on a daily or even an hourly basis depending upon process and industry.^3^

In these analytical laboratories, the samples are analyzed by technical staff by usually destroying them. After the analysis, the results are reported back to those involved in the operating system where corrective action can be taken if needed by adjusting the operating parameters accordingly. Although it is a well-established procedure in the industry for process control, the time lag between sampling, analysis, and corrective feedback makes the process less effective.^3^ The whole process itself is costly, time-consuming, inefficient, and leads to a loss of materials (destructive testing). Therefore, these plants require a fast, cost-effective, non-destructive, and preferably online analytical technique that provides onsite real-time data to save time, cost, and resources. Ultraviolet-visible (UV-Vis) spectroscopy could be a possible alternative to the current methodology for measuring the concentration of nickel, cobalt, manganese, and lithium.

UV-Vis spectroscopy is a fast, inexpensive, flexible, and non-destructive analytical technique that measures the absorbance or transmittance of light as a function of the wavelength usually in a range of 200–800 nm and is appropriate for a wide class of organic compounds and some inorganic species.^4,5^ This technique is based on the electronic transitions of molecules absorbing light which excite electrons from a lower energy orbital (highest occupied molecular orbital: HOMO) to a higher energy unoccupied orbital (lowest unoccupied molecular orbital: LUMO), where the energy of the light wavelength absorbed by the molecule is equal to the difference in the energy gap of HOMO–LUMO.^6,7^ A UV-Vis spectrometer is used to direct a light source through a sample. A detector on the opposite side records the transmitted light and the difference is calculated by the system to provide absorbance or transmittance at the corresponding wavelength. A typical graph from UV-Vis spectrometer has the baseline at the bottom with peaks pointing upward for absorbance (*A*) at the corresponding wavelengths in nanometers (nm) on the *x*-axis, where we are mostly interested in the highest intensity peak known as *λ*_max_.^6^ Although absorbance is unitless, the researcher usually uses the absorbance unit (AU) for the notation. The concentration of ions present in the solution can be calculated from the absorbance value at *λ*_max_ obtained from UV-Vis spectrometer by using the Beer–Lambert law, according to which absorbance is directly proportional to concentration and path length.^8^ The path length is the distance traveled by light from the spectrometer within the solution.

UV-Vis spectroscopy has a wide range of applications and is used in almost every field including wastewater treatment,^9,10^ characterizing colloidal nanoparticles^11,12^ and polymer impregnation,^13^ and measuring the size distribution of emulsions,^13^ and the release rates of antibiotics.^13^ It is also a powerful tool to calculate reaction rates and can monitor multiple wavelengths concurrently when coupled with mass spectroscopy.^13^ The quantification of ion concentrations using Beer–Lambert's equation is one of the applications of UV-Vis spectroscopy in chemical engineering. However, its application in battery preparation and recycling, and other related industries, to measure the concentration of various elements such as nickel, cobalt, manganese, and lithium has not been widely studied. One study did report the detection of nickel and cobalt using UV-Vis spectroscopy in a specific metal refinery in the presence of iron and copper,^3^ but no detailed analysis was performed to verify the application across different concentrations and elemental combinations. Also, the investigation of manganese and lithium in aqueous solution and the effect of their presence on other metal ions has not been reported in the literature.

In the present study, the feasibility of UV-Vis spectroscopy for onsite measurement of nickel, cobalt, manganese, and lithium was investigated. Samples of individual and different combinations of nickel, cobalt, manganese, and lithium were prepared by varying their concentrations. Measurements were carried out in a quartz cuvette cell at room temperature (25 °C) using a UV-Vis spectrometer. The effect of various parameters such as path length, concentration, solution pH, and density on the absorbance of different elements was investigated. Various mathematical relations were developed to calculate the concentration of elements using absorbance data at corresponding *λ*_max_ wavelengths. The effect of the presence of different elements on the absorbance of each was systematically analyzed and presented. Conclusions were made regarding the feasibility of UV-Vis spectroscopy application in batteries and other applicable fields for the measurement of nickel, cobalt, manganese, and lithium.

## Experimental

### Materials

All the chemicals used in the present study were of analytical grade purity and were used without any further purification. Nickel sulfate hexahydrate (NiSO_4_·6H_2_O, ≥98% pure), cobalt sulfate heptahydrate (CoSO_4_·7H_2_O, ≥99% pure), manganese sulfate monohydrate (MnSO_4_·H_2_O, ≥99% pure), lithium sulphate monohydrate (LiSO_4_·H_2_O, ≥99% pure), potassium permanganate (KMnO_4_, ≥99% pure), and potassium dichromate (K_2_Cr_2_O_7_, ≥99% pure) were purchased from Sigma Aldrich Canada (Oakville, Canada). Deionized water was produced by the Milli-Q Integral water purification system of MilliporeSigma (Merck KGaA, Darmstadt, Germany). Concentrated orthophosphoric acid (H_3_PO_4_, 85.0 wt%) and concentrated sulfuric acid (H_2_SO_4_, 95.0–98.0 wt%) were supplied by VWR International LLC (Mississauga, Ontario, Canada). Quartz cuvette cells of 10 mm and 2 mm path length (200–2500 nm scan range) with a volume of 1.2 mL (with slits) and 0.7 mL, respectively, were purchased from Lianyungang Highborn Technology Co. Ltd (Lianyungang, Lianyungang Jiangsu Province, China).

### Instrumentation

All the samples for the concentration measurements were prepared by using Hamilton Microlab 600 auto diluter system (Hamilton Company, Reno, Nevada, USA), which is more than 99% precise and independent of the liquid viscosity, vapor pressure, and temperature. For determining the metal concentrations, the samples were diluted with 5 wt% HNO_3_ before elemental analysis with ICP-OES (PerkinElmer Optima 8000 DV, Waltham, Massachusetts, USA) using the following wavelengths: Ni 231.604 nm, Co 228.616 nm, Mn 257.610 nm, and Li 610.362 nm. The absorbance of the samples was measured using a Lambda 365 UV/Vis spectrometer with a spectral range of 190–1100 nm (double beam instrument) and linear absorbance up to 3.2 (official). The density of the samples was measured using a DMA 501 density meter from Anton-Paar Canada (Saint-Laurent, Quebec, Canada) with a measurement range of 0–3 g cm^−3^ and an accuracy of 0.001 g cm^−3^.

### Calibration of the UV-Vis instrument

Prior to the sample absorbance measurement, the UV-Vis spectrometer was calibrated to ensure that the calibration on the instrument is in order and the spectrometer can be used with confidence. Two standard solutions of 0.001 M K_2_Cr_2_O_7_ and 0.0005 M KMnO_4_ were prepared using a mixture of 0.7 M H_3_PO_4_ and 1 M H_2_SO_4_ as a diluent. Mixtures containing a fixed concentration of 0.001 M K_2_Cr_2_O_7_ and 0.0005 M KMnO_4_ were prepared in different combinations as shown in Table S1.† A 10 mm path length quartz cuvette cell with quartz slits was used to measure the absorbance of prepared standards at room temperature using the UV-Vis spectrometer. The quartz slits were used to reduce the amount of sample required for each measurement without affecting the results. Before the UV-Vis measurement, the cuvette cell was first triple washed with DI water followed by washing with the actual sample to be measured three times. Any excess liquid outside the walls of the cuvette cell was cleaned using anhydrous ethanol and Kimwipes™. After that, about 1 mL of the sample was injected into the cuvette cell using a pipette and used for absorbance measurement. The process of cleaning the cuvette cell with DI water and the actual sample was repeated each time before any measurement. The baseline correction was performed without any sample in the spectrometer sample holder and the same type of 10 mm path length quartz cuvette filled with DI water was used as a reference sample. The measurements were carried out with the help of UV-Vis express software where method parameters were selected. The scan range was set in between 900 and 200 nm with a scan rate of 240 nm min^−1^ using double normal beam type and UV + Vis lamp with default light change wavelength.

### Sample preparation and UV-Vis measurement (default pH)

The process of preparing the actual samples for the UV-Vis measurement was similar to the process of preparing the standards for the instrument calibration. Based on the literature, NiSO_4_·6H_2_O, CoSO_4_·7H_2_O, MnSO_4_·H_2_O, and LiSO_4_·H_2_O were selected as the source of Ni, Co, Mn, and Li, respectively. The concentration range of individual elements and in combination was selected based on the solubility of each salt in DI water at room temperature (25 °C) that was provided by the supplier and considered the common concentration range of these elements on an industrial scale as presented in Table 1. The list of samples prepared using individual elements and in combination is presented in Table 2 along with their concentrations. To prepare a sample solution, the respective amount of metal salt/salts calculated based on solubility and the required metal concentration (see Table 2) was added to a 10 mL volumetric flask, and volume was adjusted to 10 mL using DI water. The mixture of the metal salt and DI water was ultrasonicated until a clear and homogenous solution was obtained.

**Table 1:** List of metals, source salts, and concentrations selected for the present study

Metal	Salt used	Salt solubility at 20 °C (g L^−1^)	Corresponding metal ion concentration (g L^−1^)	Selected range for metal ion concentration (g L^−1^)
Ni	NiSO_4_·6H_2_O	625	140	1 ≤ Ni ≤ 110
Co	CoSO_4_·7H_2_O	362	76	1 ≤ Co ≤ 70
Mn	MnSO_4_·H_2_O	762	248	1 ≤ Mn ≤ 70
Li	Li_2_SO_4_·H_2_O	296	32	0.1 ≤ Li ≤ 25
Ni, Co, Mn, Li				60 ≤ Ni, Co, Mn, Li ≤ 150

**Table 2:** List of samples and their corresponding concentration considered for the present study

Metal	Concentration (g L^−1^)								
**pH ∼4 (default)**									
Ni	1	5	10	30	50	70	90	110	
Co	1	5	10	20	30	40	50	60	70
Mn	1	5	10	20	30	40	50	60	70
Li	0.1	1	5	10	15	20	25		
Ni–Co	110-10	90-20	70-30	50-40	30-50	10-60			
Ni–Mn	110-10	90-20	70-30	50-40	30-50	10-60			
Co–Mn	60-10	50-20	40-30	30-40	20-50	10-60			
Ni–Co–Li	50-40-1	50-40-5	50-40-10	50-40-15	50-40-20	50-40-25			
Ni–Co–Mn	50-40-10	50-40-20	50-40-30	50-40-40	50-40-50	50-40-60			
Ni–Co–Li–Mn	50-40-1-60	50-40-5-50	50-40-10-40	50-40-15-30	50-40-20-20	50-40-25-10			

**pH ∼1 (adjusted using H**_**2**_ **SO**_**4**_ **)**									
Ni–Co	110-10	90-20	70-30	50-40	30-50	10-60			
Ni–Co–Li–Mn	50-40-1-60	50-40-5-50	50-40-10-40	50-40-15-30	50-40-20-20	50-40-25-10			

A 10 mm path length quartz cuvette cells were used to measure the absorbance of individual elements, while 2 mm path length quartz cuvette cells were used for all the samples listed in Table 2. Similar to the preparation of the standard samples, the cuvette cells were triple washed with DI water followed by washing with the actual sample to be measured three times. After that, about 1 mL or 0.65 mL of the sample to be analyzed by the UV-Vis spectrometer was injected into a 10 mm or 2 mm path length cuvette cell, respectively, using a pipette. The baseline correction was performed without any sample in the spectrometer sample holder and the same type of cuvette cell filled with DI water was used as a reference sample. The scan range in UV-Vis express software was set in between 900 and 200 nm with a scan rate of 240 nm min^−1^ using a double normal beam type and the UV + Vis lamp used a light change wavelength to 360 nm for all the samples. The light change wavelength was selected based on available information about the *λ*_max_ for cobalt and nickel to avoid any overlap with the peaks.

### Density measurement

The density of all the compositions was measured with DMA 500 density meter at 20 °C using the samples prepared for UV-Vis measurements. The instrument was first calibrated using DI water which required only 1 mL solution. Before each measurement, the measuring glass tube in the instrument was flushed with DI water twice followed by the actual sample to be measured. The solution was removed from the channel using pressurized air before slowly injecting 1 mL of the sample solution using a pipette for the density measurement. After a few minutes, the instrument provided a stable reading that was manually recorded for each sample.

### Effect of pH

To prepare a sample solution, the respective amount of metal salts calculated based on solubility and the required metal concentration (see Table 2) was added to a 10 mL volumetric flask with a small amount of DI water, and pH was adjusted to ∼1 using concentrated sulfuric acid after a complete mixing of the salts. The solution was ultrasonicated and the volume of the solution gradually increased to 10 mL by adding DI water and sulfuric acid while maintaining at pH ∼1. The samples prepared to observe the effect of pH (at pH ∼1) are listed in Table 2 along with the respective metal type and their concentrations. The sample preparation and UV-Vis measurements were carried out in the same way as described for default pH samples.

### Calibration of cuvette cell

All of the 2 mm path length cuvette cells used in the present study were triple washed with DI water followed by washing with the pure nickel or cobalt sample three times before the calibration. After that, a respective amount of nickel or cobalt sample of the same concentration was injected into each cell using a pipette before UV-Vis measurement. In the end, one cell carrying the same nickel or cobalt sample was used to investigate the reproducibility of the results by performing multiple UV-Vis measurements in the same settings. The UV-Vis spectra were acquired at the same conditions and scan range as that of all other sample measurements.

## Results and discussion

### Instrument calibration

**Fig. 1:**
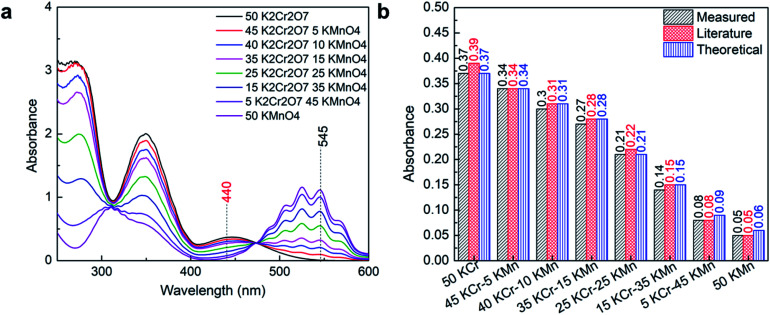
Calibration of the instrument using K_2_Cr_2_O_7_ and KMnO_4_; (a) UV-Vis spectrum of the prepared standard solutions; (b) the comparison of obtained absorbance with literature and theoretical absorbance.

The Lambda 365 UV/Vis spectrometer used for the study has many operators and is used daily. Therefore, it is necessary to check the calibration on the instrument to ensure that the responses obtained from the instrument are credible. The instrument also has internal validation tests such as wavelength accuracy and reproducibility tests, slit and filter calibration, photometric noise tests, baseline correction tests, which were automatically performed while starting the instrument for the measurement. To further ensure the data's credibility, the instrument was calibrated using 0.001 M K_2_Cr_2_O_7_ and 0.0005 M KMnO_4_. The obtained absorbance results were compared with the literature and theoretical values of absorbance for different combinations of K_2_Cr_2_O_7_ and KMnO_4_ as shown in Fig. 1. Fig. 1a shows the obtained UV-Vis absorbance spectrum of measured solutions in between 250 and 600 nm, and based on available data and literature values, peaks of 440 nm and 530 nm wavelengths were selected for the analysis and compared with literature. The experimental absorbance obtained at 440 nm matched well with the literature and theoretical absorbance as shown in Fig. 1b. This confirms the credibility of the instrument and ensures that the obtained data for different samples of nickel, cobalt, manganese, and lithium will be free from instrumental errors.

### UV-Vis analysis of the individual element

The concentration range of different elements was selected after considering various factors such as the solubility limit of the source salts in an aqueous solution, corresponding metal ion concentration, and the solution concentration at an industrial scale for battery applications. The solubility limit of an individual metal salt selected for the present study and corresponding metal ion concentration in aqueous solution are presented in Table 1 along with the lower and upper limits of individual metal ion and their combination considered for the analysis.

Multiple samples were prepared for individual elements (Ni, Co, Mn, Li) to verify the application of UV-Vis spectroscopy for the measurement of the low, medium, and high concentration solutions (Table 2). The default pH of these prepared samples was measured using a pH meter and found to be around 4.

### Effect of path length

The principle of UV-visible spectroscopy is based on the electronic transitions of organic molecules absorbing light that excite electrons from a lower energy orbital (HOMO) to a higher energy unoccupied orbital (LUMO). The light absorbance (*A*) is directly proportional to the molecular concentration (*C*), molar absorptivity (*ε*), and path length of the light traveled through the sample (*l*) as defined by Beer–Lambert law:^5,14^1
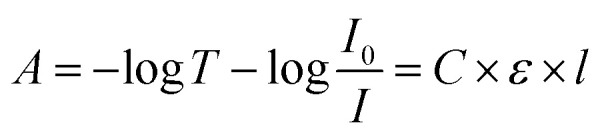
where *T* is the transmission, *I*_0_ and *I* are the intensity of the measuring beam before and after passing through the sample, respectively. Although the Beer–Lambert law can be applied to any concentration, literature shows that it follows well for the absorbance between 0.1 and 2.^3^ At absorbance 2, more than 99% percent of the incident light is absorbed by the molecules in the sample and less than 1% is transmitted to the receiver. With a further decrease in transmitted light (*i.e.*, higher than absorbance 2), the accuracy of the results becomes highly dependent on the precision of the spectrometer receiver. Therefore, it is suggested to keep the absorbance lower than 2 for a more accurate measurement of the solution concentration. As the molar absorptivity of each solution remains the same, the absorption can only be controlled by changing the path length of the light traveled through the sample to measure a fixed concentration.

### Using 10 mm path length

**Fig. 2:**
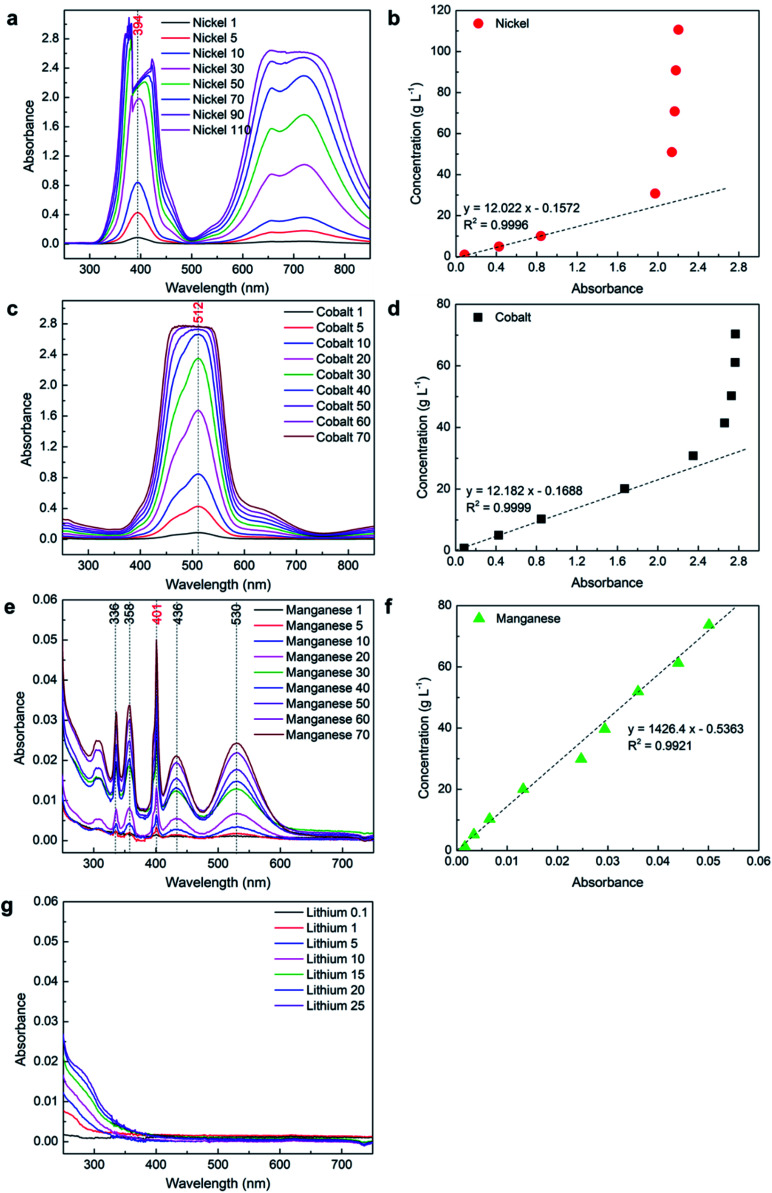
UV-Vis analysis of the samples with individual elements using a 10 mm path length cuvette cell: (a) spectrum of Ni; (b) absorbance *vs.* concentration of Ni; (c) spectrum of Co; (d) absorbance *vs.* concentration of Co; (e) spectrum of Mn; (f) absorbance *vs.* concentration of Mn; (g) spectrum of Li.

In UV-Vis spectroscopy, the absorbance of the sample is usually measured using a cuvette cell and the path length is defined by the gap between the wall of the cuvette cell perpendicular to the light travelled. In this study, a 10 mm path length quartz cuvette cell was used to measure the absorbance of Ni, Co, Mn, and Li samples of different concentrations. The obtained absorbance spectra were used to obtain the relationship between absorbance and concentration. Fig. 2 shows the UV-Vis spectrum collected for Ni, Co, Mn, and Li.

#### Nickel

The eight samples prepared using NiSO_4_·6H_2_O salt were analyzed using a 10 mm path length cuvette cell and the obtained absorbance spectrum between 250 and 850 nm is shown in Fig. 2a. The prepared samples were dark green in color given by nickel ions and the color intensity increases with an increase in the concentration of nickel ions. Three different peaks at wavelengths 394 nm, 657 nm, and 721 nm were observed from Fig. 2a and assigned to nickel only based on the literature. The highest intensity peak at 394 nm was used as *λ*_max_ for nickel and the corresponding absorbance at *λ*_max, Ni_ was used for the analysis. Fig. 2a shows that the clear peaks were observed for the solution with a nickel concentration ranging from 1 g L^−1^ to 10 g L^−1^. However, an irregularity at *λ*_max, Ni_ was observed for samples with 30 g L^−1^ or higher concentrations of nickel. This is because the absorbance of these samples reaches beyond 2 where almost all the emitted light was absorbed by the samples and the detector at the receiver end could not properly measure the transmitted light that is required to calculate the correct absorbance. These irregularities at *λ*_max, Ni,_ and the fact that Beer–Lambert law only follows well for absorbance below 2 makes the data unreliable to calculate the concentration of nickel. The absorbance values from different samples were plotted against nickel concentration obtained from ICP-OES as shown in Fig. 2b. The first three data points in Fig. 2b show a linear behavior and were used to calculate the *R*^2^ value and to define a mathematical relationship between concentration and absorbance (eqn (2)). The high *R*^2^ value of 0.9996 confirms a very linear relationship between concentration and absorbance below 2 for the pure nickel:

2 *Y* = 12.022*x* − 0.1572

where *Y* is the concentration of nickel (g L^−1^) and *x* is absorbance. Similar equations were developed for all other systems and presented in corresponding figures. A list of all the mathematical relationships developed in this study is presented in Table 6 along with their applicable absorbance and concentration range, and *R*^2^ values. The application of these equations is discussed in detail under the proposed equation section. The obtained mathematical relation and absorbance value were used to calculate the solution concentration (Conc._calculated_), which was compared with a measured concentration from ICP-OES (Conc._ICP-OES_) to find the error using eqn (3):3
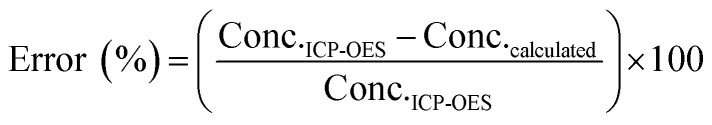


With low concentration samples of nickel when the absorbance is below 2, only a small error can be observed from Table 3, while error significantly increases at 30 g L^−1^ or beyond as the obtained absorbance data is not accurate at higher concentration. This shows that only the low concentration of nickel (<30 g L^−1^) can be measured using a 10 mm path length cuvette cell with UV-Vis spectroscopy technique. A relatively high error at 1 g L^−1^ (6%) could be due to lack of data points for the calibration curve at low concentration, and because the absorbance value was outside 0.1–2 where Beer–Lambert does not follow well.

**Table 3:** Absorbance, measured and calculated concentrations of Ni, Co, and Mn samples, and error with 10 mm path length cuvette cell

Sample	Absorbance	Measured conc. (g L^−1^)	Calculated conc.(g L^−1^)	Error (%)
Nickel 1	0.086	0.9	0.9	6.0
Nickel 5	0.427	4.9	5.0	−2.1
Nickel 10	0.842	10.0	10.0	0.5
Nickel 30	1.972	30.7	23.5	23.3
Nickel 50	2.137	50.9	25.5	49.9
Nickel 70	2.166	70.8	25.9	63.5
Nickel 90	2.178	90.8	26.0	71.3
Nickel 110	2.203	110.6	26.3	76.2
Cobalt 1	0.085	0.8	0.9	8.2
Cobalt 5	0.426	5.0	5.0	−0.1
Cobalt 10	0.848	10.3	10.2	−1.3
Cobalt 20	1.672	20.1	20.2	0.3
Cobalt 30	2.350	30.8	28.5	−8.3
Cobalt 40	2.660	41.5	32.2	−28.6
Cobalt 50	2.728	50.3	33.1	−52.1
Cobalt 60	2.765	61.1	33.5	−82.4
Cobalt 70	2.767	70.3	33.5	−109.7
Manganese 1	0.002	1.2	1.7	−45.1
Manganese 5	0.003	5.2	4.3	17.0
Manganese 10	0.007	10.3	8.7	15.2
Manganese 20	0.013	20.0	18.3	8.7
Manganese 30	0.025	29.9	34.7	−16.0
Manganese 40	0.029	39.7	41.4	−4.3
Manganese 50	0.036	51.9	50.8	2.1
Manganese 60	0.044	61.2	62.2	−1.6
Manganese 70	0.050	73.7	70.9	3.7

#### Cobalt

Similar to nickel, nine samples were prepared using CoSO_4_·7H_2_O salt were analyzed using a 10 mm path length cuvette cell, and an obtained absorbance spectrum between 250 and 850 nm is shown in Fig. 2c. The prepared samples were dark red in color given out by the cobalt ions and the intensity of color increases with an increase in the concentration of cobalt ions. In the case of cobalt, only one peak was observed from the obtained spectrum with *λ*_max, Co_ at 512 nm and assigned to cobalt as per the literature. The corresponding absorbance at *λ*_max, Co_ was used for the analysis. Similar to nickel, clear peaks were observed for the solution with the cobalt concentration ranging from 1 g L^−1^ to 20 g L^−1^ and irregularity can be observed for the samples with a higher concentration. The reason for these irregularities is the same as described for the pure nickel samples. The obtained absorbance corresponding to the *λ*_max, Co_ was used to find the mathematical relationship between absorbance and concentration as shown in Fig. 2d where the high value of *R*^2^ (0.9999) confirms a very linear relationship up to 20 g L^−1^. The error between the measured and calculated concentration (using eqn (3)) was negligible for most of the compositions up to 20 g L^−1^ except at 1 g L^−1^ (Table 3) wherein the error was relatively high (8%) due to the same reason given for the nickel samples. This shows that only the low concentration of cobalt (<30 g L^−1^) can be measured using a 10 mm path length cuvette cell with UV-Vis spectroscopy technique.

#### Manganese

The absorbance spectrum of manganese samples prepared using MnSO_4_·H_2_O salt shows multiple peaks mainly at 336 nm, 358 nm, 401 nm, 436 nm, and 530 nm, between 250 and 850 nm as shown in Fig. 2e. The prepared samples were almost colorless at a low concentration and give a light-yellow tone at a higher concentration. Due to the lack of literature related to peak position for manganese in an aqueous solution, all the obtained peaks were analyzed to calculate separate *R*^2^ values as presented in Fig. S1a (ESI†). After a careful assessment of all the peaks at different concentrations of manganese, the peak at 401 nm was selected as *λ*_max, Mn_ based on strong peak intensity and high *R*^2^ value, and used for further analysis. However, manganese shows a very low absorbance even at the highest analyzed concentration. The maximum value of absorbance for 70 g L^−1^ sample was much lower than the 0.1, which could limit the application of UV-Vis spectroscopy for the measurements of the manganese concentration even in its pure form. That being said, the absorbance values from different samples plotted against manganese concentration obtained from ICP-OES still show a linear relationship with a reasonable *R*^2^ value (0.9921) as shown in Fig. 2f. The error between the measured and calculated concentration of manganese (using eqn. (3)) presented in Table 3 shows that reasonably accurate results can be obtained for a low concentration (except 1 g L^−1^) using UV-Vis spectroscopy while a high concentration can be measured with high accuracy.

#### Lithium

The obtained lithium solutions prepared using LiSO_4_·H_2_O were completely colorless even at the highest concentration. Therefore, the absorbance spectrum of lithium samples does not show even a small peak between 250 and 850 nm as shown in Fig. 2g. Hence, the concentration of lithium can not be measured in its pure form using UV-Vis spectroscopy. Some additives may need to be added to the pure lithium solution that can combine with lithium to provide some color and make it feasible to measure lithium concentrations using UV-Vis spectroscopy. However, it may not be a practical solution at an industrial scale especially in the field of battery production.

The above results show that a 10 mm path length cuvette cell can be used to measure the concentration of pure manganese up to saturation, but can only measure the low concentration of nickel and cobalt as the absorbance value reaches beyond 2 for a high concentration. From eqn (1), it can be observed that absorbance is directly proportional to the path length. Therefore, the path length can be reduced to measure the high concentration of nickel and cobalt while keeping the absorbance below 2.

### Using 2 mm path length

To measure the high concentration of nickel and cobalt, path length was reduced five times and a 2 mm path length cuvette cell was used to measure the absorbance.

#### Nickel

**Fig. 3:**
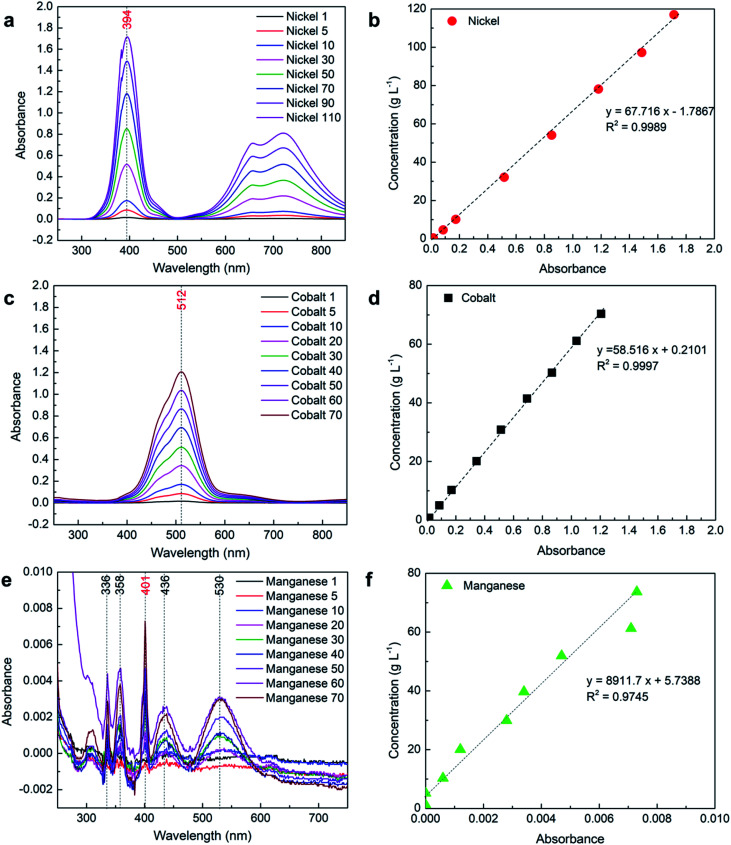
UV-Vis analysis of the samples with individual elements using a 2 mm path length cuvette cell: (a) spectrum of Ni; (b) absorbance *vs.* concentration of Ni; (c) spectrum of Co; (d) absorbance *vs.* concentration of Co; (e) spectrum of Mn; (f) absorbance *vs.* concentration of Mn.

The measurements were carried out using a 2 mm path length cuvette cell and the absorbance spectrum was collected between 250 and 850 nm. As expected, the absorbance of all the samples reduced by five times compared with the absorbance using 10 mm path length cuvette cell and lies under 2 for all measured concentrations of nickel as shown in Fig. 3a. The absorbance values corresponding to *λ*_max, Ni_ (394 nm) were plotted against measured concentration using ICP-OES in Fig. 3b show a linear relationship between concentration and absorbance of nickel for all of the samples. The error (using eqn (3)) calculated using the developed mathematical relation for different samples is presented in Table 4, which clearly shows that the concentration of the nickel can be measured by UV-Vis spectroscopy with high accuracy. Although one equation can be used to calculate the concentration of nickel from 1 to 110 g L^−1^ using a 2 mm path length cell, it is suggested to use a separate equation for absorbance below 0.2 for high accuracy at a low concentration of nickel, as presented in Table 6. A more detailed explanation regarding these equations is provided under the proposed equations section.

**Table 4:** Absorbance, measured and calculated concentrations Ni, Co, and Mn samples, and error with 2 mm path length cuvette cell

Sample	Absorbance	Measured conc. (g L^−1^)	Calculated conc.^a^ (g L^−1^)	Error (%)
Nickel 1	0.017	0.9	0.9	−2.1
Nickel 5	0.086	4.9	4.9	0.7
Nickel 10	0.174	10.0	10.0	−0.2
Nickel 30	0.516	30.7	30.5	−0.8
Nickel 50	0.851	50.9	51.9	1.9
Nickel 70	1.182	70.8	73.1	3.2
Nickel 90	1.487	90.8	92.6	2.0
Nickel 110	1.715	110.6	107.2	−3.1
Cobalt 1	0.016	0.8	0.8	−1.6
Cobalt 5	0.086	5.0	5.0	0.4
Cobalt 10	0.171	10.3	10.3	−0.1
Cobalt 20	0.344	20.1	19.9	−1.3
Cobalt 30	0.513	30.8	29.8	−3.4
Cobalt 40	0.694	41.5	40.4	−2.6
Cobalt 50	0.866	50.3	50.4	0.3
Cobalt 60	1.036	61.1	60.4	−1.2
Cobalt 70	1.207	70.3	70.4	0.1
Manganese 1	0.000	1.2	5.7	377.0
Manganese 5	0.000	5.2	5.7	10.4
Manganese 10	0.001	10.3	11.1	7.6
Manganese 20	0.001	20.0	16.4	−18.0
Manganese 30	0.003	29.9	30.7	2.6
Manganese 40	0.003	39.7	36.0	−9.2
Manganese 50	0.005	51.9	47.6	−8.3
Manganese 60	0.007	61.2	69.0	12.7
Manganese 70	0.007	73.7	70.8	−3.9

a Separate mathematical equations were used for absorbance above and below 0.2 to calculate the concentration of nickel and cobalt samples as presented in Table 6.

#### Cobalt

Similarly to nickel, the absorbance spectrum was collected between 250 and 850 nm using a 2 mm path length cuvette cell where absorbance lies below 2 for all measured concentrations of cobalt as shown in Fig. 3c. Again, the absorbance corresponding to *λ*_max, Co_ (512 nm) was plotted against measured concentration using ICP-OES in Fig. 3d shows a very linear relationship between concentration and absorbance of cobalt for all samples. The insignificant error (using eqn (3)) between the calculated and measured concentrations of cobalt shows that UV-Vis spectroscopy can be effectively used to measure the concentration of cobalt. In the case of cobalt, it is also suggested to use a separate equation for absorbance below 0.2 for high accuracy at a low concentration of cobalt (see proposed equations section).

#### Manganese

The obtained results in Fig. 3e and f show that it is more difficult to measure the presence of manganese using a 2 mm path length, especially at a low concentration. The absorbance values at low concentrations were below the detection limit of the spectrometer; therefore, it recorded a zero value even at *λ*_max, Mn_ (401 nm) for such samples as presented in Table 4. The absorbance values at different wavelengths (336, 358, 401, 436, and 530 nm) at various concentrations and corresponding *R*^2^ values are shown in Fig. S1b.† Although the absorbance values of all the samples were two orders lower than the suggested lower limit of the Beer–Lambert law (0.1) for the analysis, reasonable accurate results were obtained for the higher concentration of manganese (Table 4). Therefore, it is suggested that a 10 mm or higher path length cuvette cell be used to measure the concentration of manganese, especially for low concentrations.

The lithium samples were also measured using a 2 mm path length cuvette cell and did not show any absorbance; therefore, it is not included in the results. However, the effect of lithium presence in the absorbance of other elements of interest was analyzed along with other parameters.

### UV-Vis analysis of the combined elements

The cathodes from lithium-ion batteries are usually synthesized using multiple elements where different elements in a stoichiometric amount are combined to form a complex. Therefore, it is important to accurately measure the concentration of these elements in presence of each other. In the present study, multiple combinations of nickel, cobalt, manganese, and lithium were prepared (Table 2) and analyzed using a 2 mm path length cuvette cell to verify if the UV-Vis spectroscopy can be used to accurately measure multiple elements in a mixer.

#### Nickel–cobalt

**Fig. 4:**
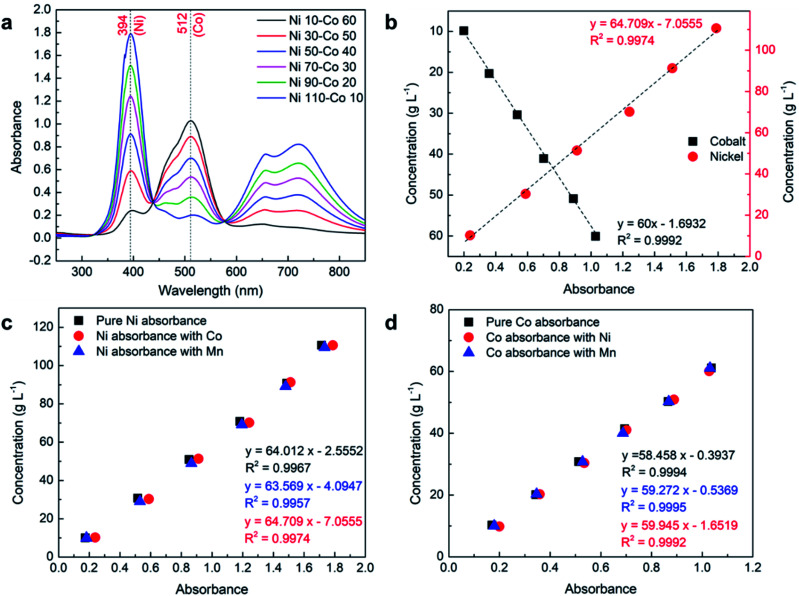
UV-Vis analysis of the samples with combined elements using 2 mm path length cuvette cell: (a) spectrum of Ni–Co; (b) absorbance *vs.* concentration of Ni–Co; (c) effect of Co and Mn on Ni absorbance; (d) effect of Ni and Mn presence on Co absorbance.

Six samples of nickel and cobalt mixtures were prepared by gradually increasing the concentration of one element and decreasing the other as presented in Table 2. The absorbance spectrum of the samples collected using 2 mm path length cuvette cell presented in Fig. 4a shows that both cobalt and nickel have clear separate peaks with original *λ*_max_ at 394 nm and 512 nm, respectively, which confirm that they do not chemically interact with each other or affect their *λ*_max_ position when mixed in an aqueous solution. The absorbance values of cobalt and nickel plotted against measured concentrations in Fig. 4b show that both the elements still have a very linear relationship between concentration and absorbance. The insignificant error (using eqn (3)) between the measured and calculated concentrations of nickel and cobalt presented in Table 5 shows that both elements can be measured with high accuracy even in the presence of each other.

**Table 5:** Absorbance, measured, and calculated concentration of Ni and Co mixture, and percentage error with 2 mm path length cuvette cell^a^

Sample	Absorbance (Ni)	Measured conc. (g L^−1^)	Calculated conc. (g L^−1^)	Error (Ni) (%)	Absorbance (Co)	Measured conc. (g L^−1^)	Calculated conc. (g L^−1^)	Error (Co) (%)
Ni 10–Co 60	0.239	10.2	9.8	3.9	1.028	60.1	60.0	0.2
Ni 30–Co 50	0.588	30.3	31.0	−2.2	0.889	50.9	51.6	−1.5
Ni 50–Co 40	0.911	51.4	51.9	−1.1	0.701	41.1	40.4	1.7
Ni 70–Co 30	1.242	70.1	73.3	−4.5	0.535	30.4	30.4	−0.2
Ni 90–Co 20	1.511	91.3	90.7	0.6	0.359	20.3	19.8	2.1
Ni 110–Co 10	1.788	110.6	108.6	1.8	0.200	9.8	10.3	−4.8

a Concentration was calculated using the separate equations developed for the combination of Ni–Co presented in Table 6.

#### Nickel–manganese

Fig. S2a† shows the absorbance spectrum of nickel and manganese samples that were prepared by gradually increasing the concentration of one element and decreasing the other. The obtained spectrum shows only the peaks corresponding to nickel while the peaks associated with manganese are missing. This happens because the *λ*_max, Mn_ (401) is very close to *λ*_max, Ni_ (394); therefore, they overlap with each other. At the same time, the absorbance of manganese is a few orders lower than the nickel absorbance, so all of the peaks are covered under the background of the nickel peaks. This shows that the concentration of manganese cannot be measured in the presence of nickel. The absorbance values of nickel plotted against measured concentration in Fig. S2b† still show a very linear relationship, and error (using eqn (3)) between measured and calculated concentration remained insignificant as presented in Table S2.†

#### Cobalt–manganese

Even though *λ*_max, Co_ (512) was not very close to *λ*_max, Mn_ (401), only cobalt peaks were observed from the absorbance spectrum of cobalt and manganese mixtures as shown in Fig. S3a.† Again, this is because the absorbance of manganese is a few orders lower than the cobalt absorbance, so all the peaks are covered under the background of the cobalt peak. This means that the concentration of manganese cannot be measured in the presence of both cobalt and nickel. The absorbance values of cobalt plotted against measured concentration in Fig. S3b† still show a very linear relationship, and the error between measured and calculated concentration remained insignificant as presented in Table S3.†

Although the concentration of manganese cannot be determined in presence of nickel or cobalt or both, its presence may affect the absorbance of cobalt and nickel. Also, even though both nickel and cobalt are chemically inert together in an aqueous solution, their presence may affect each other absorbance. Therefore, a detailed analysis was performed to identify the effect of nickel, cobalt, and manganese on each other. Fig. 4c and d show the comparison of absorbance of pure nickel and cobalt with the presence of each other and manganese, respectively. The absorbance of each element at the *λ*_max_ of nickel and cobalt at different concentrations was investigated. A detailed analysis shows that the presence of nickel and cobalt have a small effect on each other's absorbance. This effect is not due to any chemical interactions between the elements but because of a small absorbance of these elements under the same wavelength of the other element's *λ*_max_ region which can be observed from their absorbance spectrum in pure form (see Fig. 3a and c). The effect is comparatively more significant on nickel with the presence of cobalt than otherwise due to the fact that cobalt absorbance at *λ*_max, Ni_ is significantly higher than nickel absorbance at *λ*_max, Co_. Although the effect of these absorbances on each other's *λ*_max_ is not substantial, separately developed equations (Fig. 4c and d) are suggested for use in the calculation if the presence of a second element is detected from the absorbance spectrum (see proposed equation section). A similar analysis was performed to study the effect of the presence of manganese on the absorbance of cobalt and nickel. The detailed analysis shows that even though *λ*_max, Mn_ (401) roughly overlaps with *λ*_max, Ni_ (394), the effects are almost insignificant due to the very low absorbance of manganese compared to nickel as presented in Fig. 4c. Similarly, the effect of the presence of manganese is also insignificant on cobalt absorbance as presented in Fig. 4d. In the next step, the effect of manganese was systematically analyzed on a mixture of nickel–cobalt along with the presence of lithium.

### Effect of manganese and lithium on nickel–cobalt absorbance

The previous results show that the concentration of lithium cannot be determined using UV-Vis, while the presence of manganese can be determined in its pure form. However, the presence of these two elements individually or together with the nickel–cobalt mixture may affect their absorbance. Therefore, a systematic study was carried out to determine the effect of manganese and lithium on nickel–cobalt absorbance, where a mid-range concentration of nickel (50 g L^−1^) and cobalt (40 g L^−1^) was selected for the analysis. Different samples were prepared with a fixed concentration of nickel and cobalt while gradually increasing the concentrations of manganese and lithium, individually from 1 g L^−1^ to close to saturation as presented in Table 2.

**Fig. 5:**
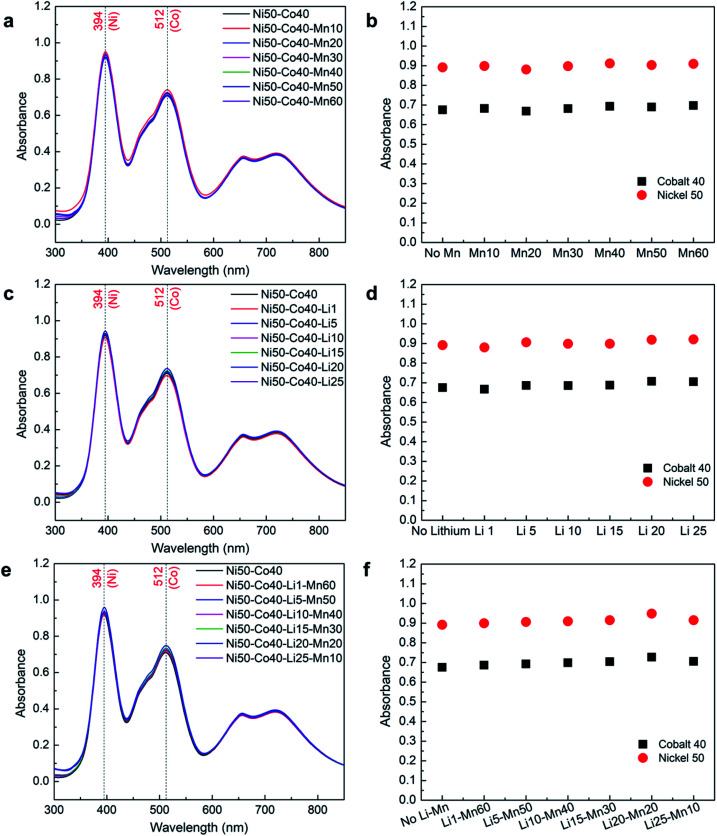
UV-Vis analysis to observe the effect of Li and Mn on Ni–Co absorbance: (a) spectrum of Ni–Co with Mn; (b) normalized absorbance of Ni–Co with Mn; (c) spectrum of Ni–Co with Li; (d) normalized absorbance of Ni–Co with Li; (e) spectrum of Ni–Co with Li and Mn; (f) normalized absorbance of Ni–Co with Li and Mn.

The absorbance spectra obtained from samples with a fixed concentration of nickel–cobalt with varying concentrations of manganese and lithium in Fig. 5a and c, respectively, show that all the spectra follow each other with very small variations. These variations could be due to differences in the concentration of nickel and cobalt among different samples as each sample was separately prepared. To verify the effect of manganese and lithium, the obtained absorbance corresponding to *λ*_max_ of nickel and cobalt were normalized using their measured concentrations from ICP-OES and compared in Fig. 5b and d, respectively. The comparisons show that there is no or insignificant effect of manganese or lithium presence on the absorbance of nickel and cobalt mixture. The average error between the absorbance values of nickel and cobalt with and without manganese and lithium remains lower than 2% across different samples.

A similar analysis was performed to observe the combined effect of manganese and lithium on cobalt and nickel absorbance as shown in Fig. 5e and f, where the concentrations of cobalt and nickel were fixed, and the concentrations of manganese and lithium were gradually varied. As expected, the normalized absorbance values corresponding to *λ*_max_ of nickel and cobalt with and without manganese and lithium remain the same as shown in Fig. 5f. This confirms that there is no or insignificant effect of manganese and lithium when presented together on the absorbance of nickel and cobalt mixture. In this case, the average error among the absorbance of the different samples was less than 1%. These results confirm that the concentrations of nickel and cobalt can be measured with high accuracy through UV-Vis spectroscopy even in the presence of manganese and lithium.

### Density measurements and their effect on absorbance

The density of all the prepared samples was measured at 20 °C using a DMA 500 density meter to study the relationship with the absorbance of different metals as presented in Table S4.† The density of DI water used to prepare the samples was also measured to calibrate the density meter. The density of the individual element solution plotted against their concentrations in Fig. S4† shows that density linearly increases with increasing concentration. It is also observed that the density of nickel, cobalt, and manganese follows the same trend, while lithium solution has a comparatively higher density relative to its concentration. A detailed comparison of the obtained density results and absorbance shows that they have a very linear relationship with nickel, cobalt, and manganese. Since the density of nickel, cobalt, and manganese follows the same trend with respect to their concentrations and has a linear relationship with absorbance, it is unlikely to have any effect on absorbance when mixed. Although lithium shows different trends in terms of density, no effect is expected as it does not show any absorbance, which was confirmed previously.

### Effect of pH on absorbance

**Fig. 6:**
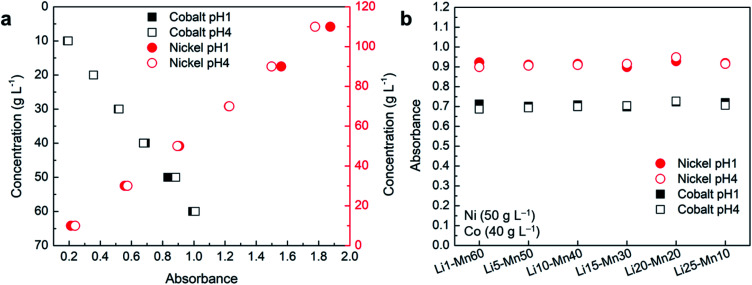
Comparison of absorbance at pH 1 and 4 for the samples containing: (a) Ni–Co; (b) Ni–Co–Mn–Li.

It is important to know if a change in pH affects the absorbance of different elements, as the solution pH in battery and other applicable industries is varied during the process for several reasons. As the default pH of the prepared samples of nickel, cobalt, manganese, and lithium was around pH 4, samples were prepared at pH 1 by using concentrated H_2_SO_4_ as presented in Table 2. The absorbance of these samples was measured and the spectrum was compared with the samples at default pH as shown in Fig. S5.† The comparisons of these spectra at pH 1 and pH 4 show no difference in peak position and follow each other well across all compositions. The minor difference could be due to a variation in the concentrations of nickel and cobalt among the samples as they were prepared separately. The comparison of absorbance corresponding to *λ*_max_ of nickel and cobalt at different pH for samples prepared by varying the concentration of these elements shows that the change in pH has almost no effect on absorbance (Fig. 6a). A small difference in the absorbance of nickel at different pH for high concentration samples could be due to variation in the path length of the cuvette cell as multiple cuvettes were used for the measurements.

A similar analysis was performed with the samples using the fixed concentration of nickel (50 g L^−1^) and cobalt (40 g L^−1^), and varying the concentration of manganese and lithium to observe the effect of pH change as shown in Fig. S5b and 6b.† The absorbance analysis at a corresponding *λ*_max_ of nickel and cobalt shows no or insignificant effect of pH change on absorbance as shown in Fig. 6b. These results confirm that changes in pH have no or insignificant effect on absorbance regardless of their concentrations and elemental composition as the average error remains lower than 1% across all the samples. Hence, UV-Vis spectroscopy can be effectively used to measure the concentration of nickel and cobalt at different pH.

### Cuvette calibration and repeatability

Five cells of 2 mm path length cells were used in random order for the UV-Vis measurement of different samples including the one used as the reference cell containing pure DI water. Although these cuvette cells were made of the same material (quartz), supplied by the same company, and were rated for the same path length (2 mm), there could be minor differences in the material and path length that could affect the absorbance significantly especially at low concentration. Therefore, these cells were calibrated using pure nickel (50 g L^−1^) and cobalt (40 g L^−1^) samples separately in the same order. The UV-Vis measurements were carried out by using the same sample with each cell and results are compared in Table S5† where the cell with the lowest absorbance was considered for reference and assumed to have an exact 2 mm path length. It can be observed from Table S5† that all the cells show slightly different absorbance when the same sample was used for the measurement. This shows that these cells have a small difference in their path length which leads to a slight difference in absorbance as both are directly proportional. Cell 2 shows the highest absorbance with a difference of 1.3% from the reference both in the case of nickel and cobalt with a corresponding path length of 2.026 mm. This proves the hypothesis considered earlier that a difference in path length of the cells could lead to error observed in some of the samples. Although the maximum difference in path length is only 1.3%, it could lead to higher error in a calculated concentration obtained from developed mathematical relations depending upon the concentration of the sample used with sample 2.

Cell 4 was also used to investigate the repeatability of the measurements from the UV-Vis spectrometer where four spectra were collected for the same sample and at the same measuring conditions. The obtained results presented in the Table S5† show a minor difference between the absorbance across four different cycles. These differences are mainly related to the accuracy of the UV-Vis spectrometer used in this study as there is no other factor influencing the absorbance. Therefore, the accuracy of the instrument may also be contributing to the small error observed in the sample measurements.

### Proposed equations

After a detailed analysis of the obtained results, a list of mathematical relationships was developed and presented in Table 6 for different scenarios and elements of interest. These equations can be directly used by researchers to calculate the concentration of different elements using the obtained absorbance spectrum. Although it is suggested to use a 2 mm path length cuvette for measuring nickel and cobalt concentrations, eqn (4) and (5) in Table 6 can be used to calculate the low concentration of nickel and cobalt, respectively, while using a 10 mm path cuvette cell. For pure manganese, it is strongly suggested to use a 10 mm path length cuvette for high accuracy and the concentration can be measured using eqn (6). For the measurements of pure nickel and cobalt using a 2 mm path length cuvette, separate equations were developed for absorbance above and below 0.2 to reduce the calculation error at low concentrations. The eqn (7) (Table 6) can be used to calculate the concentration of nickel if obtained absorbance at corresponding *λ*_max, Ni_ is less than 0.2, and eqn (8) can be used for absorbance above 0.2. Similarly, eqn (11) and (12) can be used to calculate the concentration of cobalt for absorbance below and above 0.2, respectively. Although it may be possible to have one equation for both low and high concentrations of each element by including several additional data points in the calibration curve especially at the low concentration region, the appropriate equation can be easily selected based on the absorbance and provide accurate results.

**Table 6:** Obtained mathematical relations and *R*^2^ values corresponding to different absorbance and concentration range for various combinations of elements

Element	Path length (mm)	Absorbance range	Concentration range (g L^−1^)	Mathematical relation	*R* ^2^ value
Ni	10	0.086-0.842	1-10	*y* = 12.022*x* − 0.1572 (4)	0.9996
Co	10	0.085-1.672	1-20	*y* = 12.182*x* − 0.1688 (5)	0.9999
Mn	10	0.002-0.050	1-70	*y* = 1426.4*x* − 0.5363 (6)	0.9921
Ni	2	0.017-0.174	1-10	*y* = 57.557*x* − 0.0337 (7)	1
Ni	2	0.174-1.715	10-110	*y* = 64.012*x* − 2.5552 (8)	0.9967
Ni–Mn (Ni)^a^	2	0.181-1.734	10-110	*y* = 63.569*x* − 4.0947 (9)	0.9957
Ni–Co (Ni)	2	0.239-1.788	10-110	*y* = 64.709*x* − 7.0555 (10)	0.9974
Co	2	0.016-0.171	1-10	*y* = 61.584*x* − 0.2106 (11)	1
Co	2	0.171-1.207	10-70	*y* = 58.097*x* + 5762 (12)	0.9995
Co–Mn (Co)^a^	2	0.179-1.031	10-60	*y* = 59.287*x* − 0.5312 (13)	0.9995
Co–Ni (Co)	2	0.200-1.028	10-60	*y* = 60*x* − 1.6932 (14)	0.9992

a As the presence of manganese has negligible or no effect on absorbance on Ni or Co, mathematical relation corresponding to pure Ni or Co can be used in presence of manganese.

As discussed earlier, even though the studied elements do not chemically interact with each other, their presence with another element can influence the corresponding absorbance depending upon their absorbance at *λ*_max_ of the other element. Therefore, if a cobalt peak is observed from the absorbance spectrum of nickel and *vice versa*, it is suggested to use eqn (10) and (14) instead of the equation for pure elements to calculate the concentration of nickel and cobalt, respectively, for more accurate results. The presence of manganese has no or insignificant effect on the absorbance spectrum of nickel and cobalt due to its extremely low absorbance regardless of its concentration. Although separate equations are provided to calculate the concentration of nickel (eqn (9)) or cobalt (eqn (13)) in the presence of manganese, the equation developed for pure nickel and cobalt can be used without any significant error, if the presence of manganese is unknown.

## Conclusions

In this study, the feasibility of the onsite measurement of nickel, cobalt, manganese, and lithium in an aqueous solution using UV-Vis spectroscopy was investigated for battery material processing and other applicable industries. Several samples were prepared and analyzed at room temperature (25 °C) using quartz cuvette cell with Lambda 365 UV/Vis spectrometer. The effect of various parameters such as path length, element concentration, pH, and density has been investigated to validate the applicability. The measurement of different elements in a multi-ion solution and their effect on precise measurement of each other concentration was investigated. Based on the obtained results, the following conclusions are made:

• The UV-Vis spectroscopy can be effectively used to measure the concentration of nickel and cobalt in a solution up to saturation with high accuracy using a 2 mm path length cuvette, but limited to around 20 g L^−1^ concentration with a 10 mm path length cuvette cell.

• The presence of nickel and cobalt has a small effect on each other's absorbance; therefore, the use of separate equations is suggested if the presence of other elements is detected.

• The presence of manganese and lithium has no or insignificant effect on the absorbance of nickel and cobalt.

• A change in solution pH has no or insignificant effect on the absorbance of nickel and cobalt with or without the presence of manganese and lithium.

• It is possible to measure the concentration of pure manganese with a 10 mm path length with reasonable accuracy, but not feasible with the presence of nickel or cobalt. A 2 mm path length cuvette cell can also be used to measure a higher concentration of manganese (>10 g L^−1^).

• It is not possible to measure pure lithium concentration using the UV-Vis spectroscopy technique as it does not show any absorbance regardless of its concentration.

Overall, the UV-Vis spectroscopy can be used for online measurement of nickel and cobalt concentration up to saturation for battery and other applicable industries using a 2 mm path length cuvette cell in any solution pH regardless of the presence of manganese and lithium. The concentration of manganese can be measured with reasonable accuracy with 10 mm path length up to saturation, but lithium detection is not possible regardless of its concentration.

## Author contributions

Monu Malik: conceptualization, data curation, formal analysis, investigation, methodology, visualization, writing – original draft, writing – review & editing. Ka Ho Chan: data curation and analysis, methodology, visualization, writing – review & editing. Gisele Azimi: conceptualization, funding acquisition, project administration, resource, supervision, visualization, writing – review & editing.

## Conflicts of interest

The authors declare no conflicts of interest.

## Supplementary Material

RA-011-D1RA03962H-s001
