# Supplementary material for: Quantification of nickel, cobalt, and manganese concentration using ultraviolet-visible spectroscopy
Source: RSC Adv. 2021 Aug 19;11(45):28014–28. doi: 10.1039/d1ra03962h (PMC9038011; doi:10.1039/d1ra03962h)
Supplement: RA-011-D1RA03962H-s001 [file RA-011-D1RA03962H-s001.pdf]

Electronic Supplementary Material (ESI) for RSC Advances.
This journal is © The Royal Society of Chemistry 2021

May 20, 2021

Supplementary Material (ESI) for RSC Advances

Quantification of Nickel, Cobalt, and Manganese Concentration using Ultraviolet-Visible Spectroscopy

Monu Malik,[a] Ka Ho Chan,[a] Gisele Azimi[a,b*]

[a] Department of Chemical Engineering and Applied Chemistry, University of Toronto, 200 College Street, Toronto, Ontario, M5S 3E5, Canada

[b] Department of Materials Science and Engineering, University of Toronto, 184 College Street, Toronto, Ontario, M5S 3E4, Canada

*Corresponding Author: g.azimi@utoronto.ca, +1 (416) 946-7568

SUPPLEMENTARY INFORMATION

- Number of Figures: 5
- Number of Tables: 5
- Number of Pages: 6

Table S1. Details of standard samples prepared for the calibration of the equipment using $K_2Cr_2O_7$ and $KMnO_4$.

**Sample**	**0.001 M $K_2Cr_2O_7$ (mL)**	**0.0005 M $KMnO_4$ (mL)**
50KCr	50	0
45KCr-5KMn	45	5
40KCr-10KMn	40	10
35KCr-15KMn	35	15
25KCr-25KMn	25	25
15KCr-35KMn	15	35
5KCr-45KMn	5	45
50KMn	0	50

KCr - $K_2Cr_2O_7$
KMn - $KMnO_4$

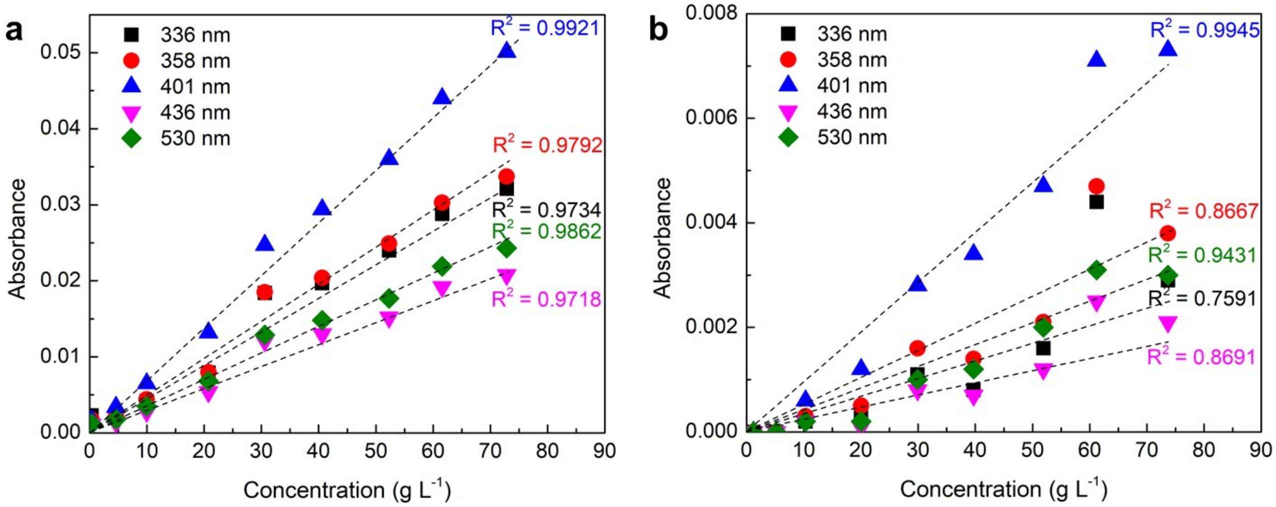

Figure S1. Absorbance of pure manganese at different wavelengths across different concentrations using (a) 10 mm and, (b) 2 mm path length quartz cuvette cells.

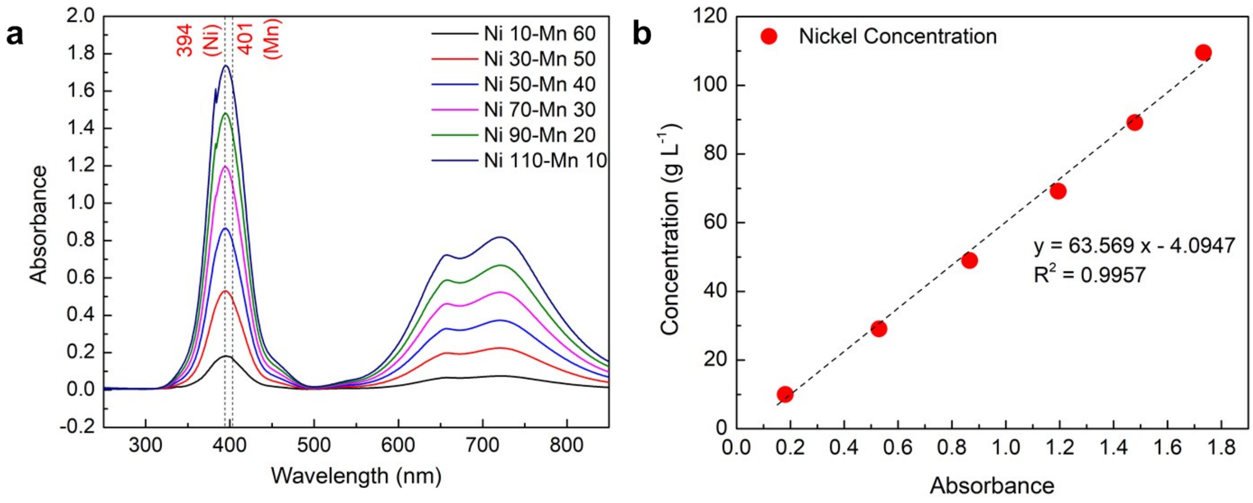

Figure S2. UV-Vis analysis of the samples with combined elements using 2 mm path length cuvette; (a) spectrum of Ni-Mn, (b) absorbance vs concentration of Ni-Mn.

Table S2. Absorbance, measured and calculated concentration of Ni-Mn mixture, and percentage error with 2 mm path length cuvette cell.

Sample	Absorbance	Measured conc. (g L^{-1})	Calculated conc. (g L^{-1})*	Error (%)
Ni 10-Mn 60	0.1811	9.9	7.4	25.5
Ni 30-Mn 50	0.5297	29.1	29.6	-1.7
Ni 50-Mn 40	0.8657	49.0	50.9	-4.0
Ni 70-Mn 30	1.1945	69.1	71.8	-3.9
Ni 90-Mn 20	1.4792	89.1	89.9	-0.9
Ni 110-Mn 10	1.7337	109.5	106.1	3.1

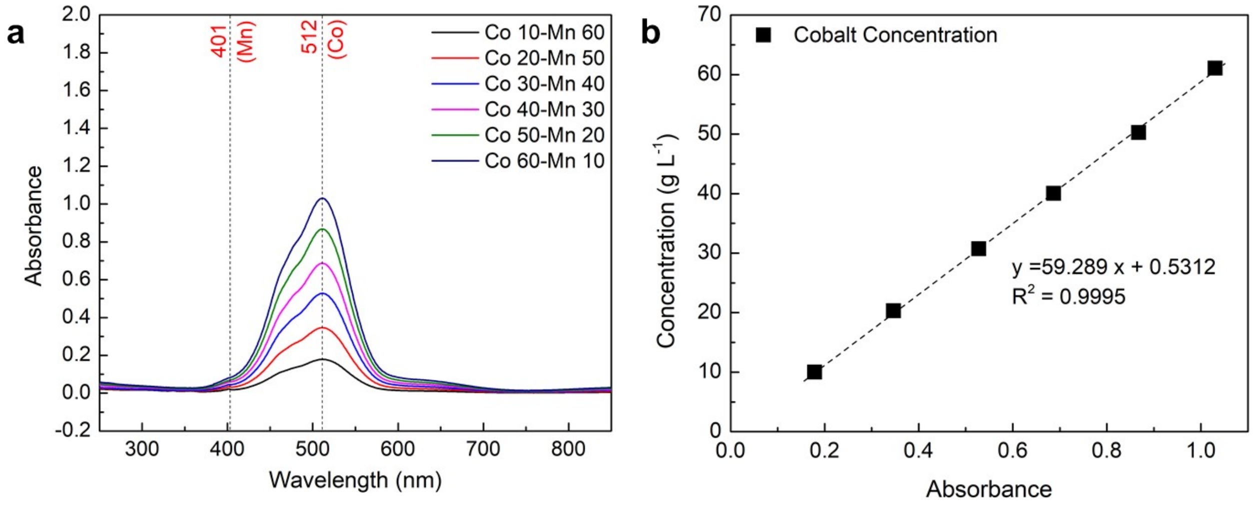

Figure S3. UV-Vis analysis of the samples with combined elements using 2 mm path length cuvette; (a) spectrum of Co-Mn, (b) absorbance vs concentration of Co-Mn.

Table S3. Absorbance, measured and calculated concentration of Co-Mn mixture, and percentage error with 2 mm path length cuvette cell.

Sample	Absorbance	Measured conc. (g L^{-1})	Calculated conc. (g L^{-1})*	Error (%)
Co 10-Mn 60	0.1789	10.0	10.1	-0.7
Co 20-Mn 60	0.3463	20.3	20.0	1.5
Co 30-Mn 40	0.5275	30.8	30.7	0.0
Co 40-Mn 30	0.6868	40.1	40.2	-0.3
Co 50-Mn 20	0.8675	50.3	50.9	-1.3
Co 60-Mn 10	1.0304	61.1	60.6	0.9

Table S4. Density of all the prepared sample and DI water at 20 °C measured using DMA 500 density meter.

Sample	Density ($g\ cm^{-3}$)	Sample	Density ($g\ cm^{-3}$)	Sample	Density ($g\ cm^{-3}$)
Ni 1	1.0012	Li 0.1	0.9990	Ni 50-Co 40-Li 1	1.2360
Ni 5	1.0123	Li 1	1.0053	Ni 50-Co 40-Li 5	1.2582
Ni 10	1.0262	Li 5	1.0319	Ni 50-Co 40-Li 10	1.2862
Ni 30	1.0797	Li 10	1.0637	Ni 50-Co 40-Li 15	1.3124
Ni 50	1.1326	Li 15	1.0943	Ni 50-Co 40-Li 20	1.3376
Ni 70	1.1831	Li 20	1.1239	Ni 50-Co 40-Li 25	1.3627
Ni 90	1.2343	Li 25	1.1531	Ni 50-Co 40-Mn 10	1.2546
Ni 110	1.2811	Ni 10-Co 60	1.1808	Ni 50-Co 40-Mn 20	1.2774
Co 1	1.0012	Ni 30-Co 50	1.2054	Ni 50-Co 40-Mn 30	1.3013
Co 5	1.0113	Ni 50-Co 40	1.2298	Ni 50-Co 40-Mn 40	1.3242
Co 10	1.0261	Ni 70-Co 30	1.2544	Ni 50-Co 40-Mn 50	1.3441
Co 20	1.0530	Ni 90-Co 20	1.2776	Ni 50-Co 40-Mn 60	1.3696
Co 30	1.0796	Ni 110-Co 10	1.3016	Ni 50-Co 40-Li 1-Mn 60	1.3733
Co 40	1.1060	Ni 10-Mn 60	1.1749	Ni 50-Co 40-Li 5-Mn 50	1.3715
Co 50	1.1317	Ni 30-Mn 50	1.2012	Ni 50-Co 40-Li 10-Mn 40	1.3735
Co 60	1.1574	Ni 50-Mn 40	1.2267	Ni 50-Co 40-Li 15-Mn 30	1.3790
Co 70	1.1821	Ni 70-Mn 30	1.2516	Ni 50-Co 40-Li 20-Mn 20	1.3839
Mn 1	1.0009	Ni 90-Mn 20	1.2757	Ni 50-Co 40-Li 25-Mn 10	1.3832
Mn 5	1.0117	Ni 110-Mn 10	1.3031		
Mn 10	1.0255	Co 10-Mn 60	1.1761		
Mn 20	1.0514	Co 20-Mn 60	1.1758		
Mn 30	1.0778	Co 30-Mn 40	1.1779		
Mn 40	1.1028	Co 40-Mn 30	1.1783		
Mn 50	1.1278	Co 50-Mn 20	1.1781		
Mn 60	1.1525	Co 60-Mn 10	1.1804		
Mn 70	1.1766	Ni 50-Co 40	1.2308		
DI Water	0.9979	DI Water*	0.9983	DI Water*	0.9983

The different color shade in the column means that samples were analyzed at different time intervals.

*The minor difference in the density of DI water is because measurements were performed at time intervals using separate DI water and used for the calibration of the instrument.

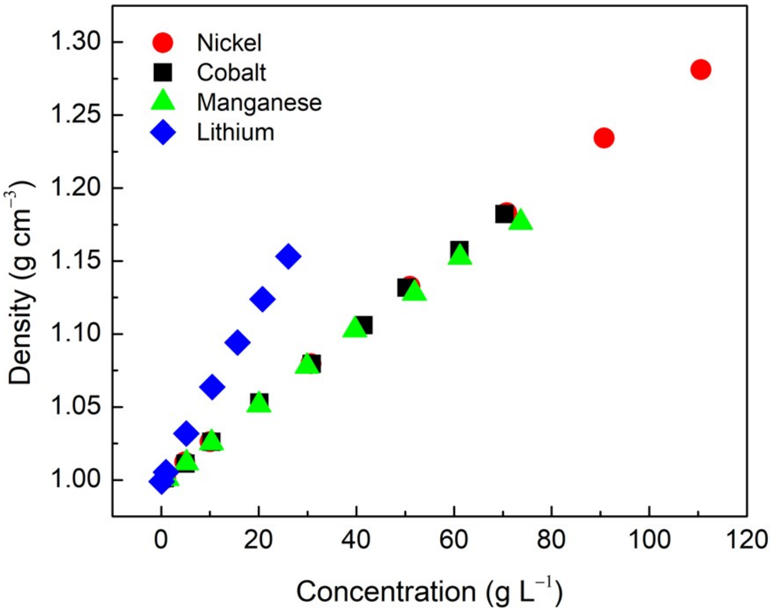

Figure S4. The relation between the density of the Ni, Co, Mn, and Li solution with their concentration.

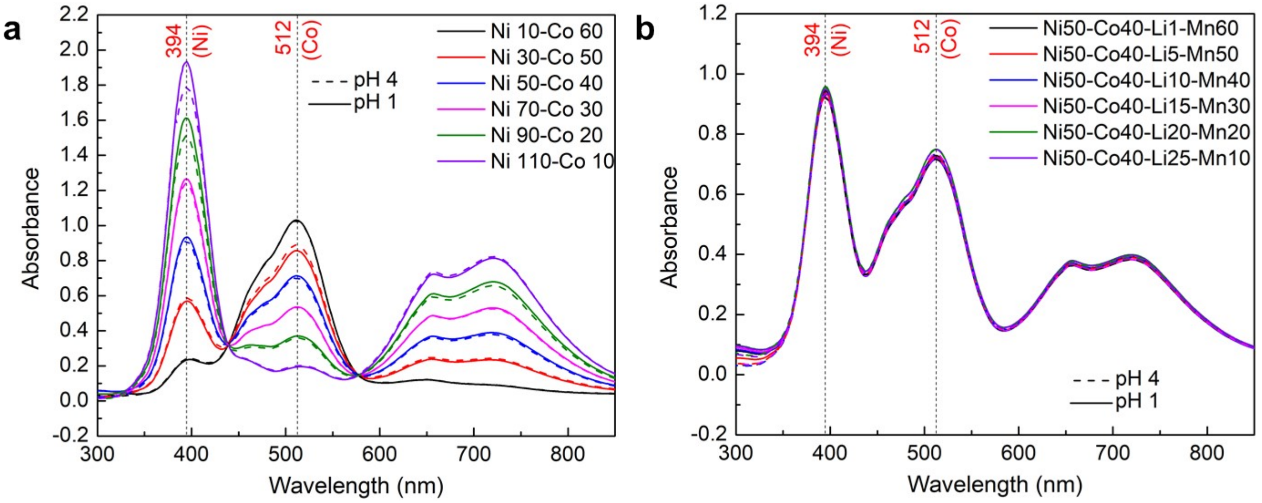

Figure S5. Comparison of absorbance spectrum collected with 2 mm path length cuvette at pH 1 and 4 for the samples containing; (a) Ni-Co, (b) Ni-Co-Mn-Li.

Table S5. Calibration and repeatability results from four cuvette cells used in the present study.

Cuvette cell	Absorbance	Difference (%)*	Path length (mm)	Cycle (Cell 4)	Absorbance	Difference (%)**
With Nickel Sample (50 g L^{-1})						
Cell 1	0.8971	0.3	2.005	Cycle 1	0.8947	0.0
Cell 2	0.9066	1.3	2.027	Cycle 2	0.8967	0.2
Cell 3	0.9029	0.9	2.018	Cycle 3	0.8977	0.3
Cell 4	0.8947	0.0	2.000	Cycle 4	0.8971	0.3
With Cobalt Sample (40 g L^{-1})						
Cell 1	0.7216	0.0	2.001	Cycle 1	0.7213	0.0
Cell 2	0.7309	1.3	2.026	Cycle 2	0.7249	0.5
Cell 3	0.7244	0.4	2.009	Cycle 3	0.7219	0.1
Cell 4	0.7213	0.0	2.000	Cycle 4	0.7249	0.5

*Cell with the lowest absorbance was considered as reference and assume to have 2 mm path length for calculations.

**Cycle with the lowest absorbance was considered as a reference for calculations.
